# Implantology and Periodontal Disease: The Panacea to Problem Solving?

**DOI:** 10.2174/1874210601711010460

**Published:** 2017-08-30

**Authors:** Giovanni Matarese, Luca Ramaglia, Luca Fiorillo, Gabriele Cervino, Floriana Lauritano, Gaetano Isola

**Affiliations:** 1 Department of Biomedical, Odontostomatological Sciences and of Morphological and Functional Images, School of Dentistry, University of Messina, Messina, Italy; 2 Department of Neurosciences, Reproductive and Odontostomatological Sciences, School of Medicine, University of Naples “Federico II”, Naples, Italy

**Keywords:** Periodontal disease, Implantology, Periodontium, Epidemiology, Peri-Implantitis, Implant-prosthetic therapy

## Abstract

**Introduction:**

The specialty of periodontology has changed dramatically in recent years. With the long-standing goal of retaining teeth in a functional and esthetical state, the periodontology has developed a high level of expertise in the regeneration of bone and connective tissues that support the teeth. However, periodontists have also joined maxillofacial surgeons as the primary providers of implant surgery.

**Methods:**

The tremendous innovations of periodontists induced also by the marketplace resulted in predictable periodontal treatment outcomes for most patients by the implants led to a dramatically different marketplace in which many patients with periodontitis can be treated by the implants rather than the traditional periodontal treatment.

**Results:**

The aim of this article is to focus on the innovator’s dilemma for periodontists today is that key elements of our rewarding contributions to dentistry in recent decades are unlikely to be part of a strong and rewarding future for the profession.

**Conclusions:**

With the intriguing role of the personalized medicine approach that integrates genomic and clinical information to predict a possible predisposition, we do not suggest a reduced role for periodontists in dental implant surgery but rather a more prominent role in complex cases to achieve surgical implant needs and proper reconstruction and long-term maintenance of the patient’s health.

## 1 ARTICLE

During the meeting of the European Federation of Periodontology “Europerio 8” in London on June 2015, Dr Niklaus P. Lang presented the Keynote Lecture “Insights of Periodontal Therapy” in which the future direction of periodontology was analyzed. The main subject of Prof. Lang’s lecture was the future of research and clinical practice in periodontics and implantology. The question that was raised for the auditorium during the lecture, was the following: Are we slightly changing our mind as periodontists to become implantologists? And how is this translated in the approach of the periodontal treatment plan?

## 2 THE ROLE OF PERIODONTOLOGY

During the last few years, several conferences on the periodontal field have promoted implant techniques, the placement of implants and their biomaterials more and more in order to obtain better long-term implant osseointegration. In addition, more and more space has been given to continuing education training times to different implant techniques. Even the main sponsorship of major national and international conferences has been carried out by implant and biomaterial industries, so this could significantly influence the content of the conferences themselves.

At the Europerio 7 (2012) congress, 60% of the main sponsors were represented by industries producing implant equipment and almost 30% of the main sessions of the congress was based implant topics [1, 2]. In the congress of the last few years of the American Academy of Periodontology (AAP), just under half of the companies present at the conference were companies producing implants and at Europerio 8 almost all of the main sponsors, especially during the lunch session, were implant manufacturers that advertise their new products with great emphasis [3].

Who is actually involved in determining the content of these meetings? Are these the most attractive and current issues for research and for clinical practice or are they indirectly induced by major sponsors? Is periodontics increasingly turning into implant therapy?

Referring to the guidelines of the AAP, which define the role of the periodontist as a clinical “specialist in the correct therapy and prevention of periodontal diseases and in the placement and maintenance of dental implants”, as we actually are, how many resources are we spending on our primary task, namely clinical risk stratification, prevention of risk factors useful to the maintenance of periodontal disease, trying to preserve the natural teeth of the patient for as long as possible. So, are we trying to offer, especially with the latest methods for stratifying the risk of tooth loss, the best care ever, or are we slowly approaching periodontal problems with an increasingly “implant mind” point of view? Moreover, the age of disease onset should also be kept in mind. In fact, periodontitis is an inflammatory disease [4, 5] typically in the adult different from implant and peri implant diseases and which seems to be a disease in which the onset is still not well defined at this time and the present epidemiological studies are not providing more precise data on it [6, 7].

A recent study has also shown that, in the absence of a systematic form of periodontal supportive 10-year therapy, patients undergoing implant therapy may develop evident bone and the peri-implant soft tissue lesions [6]. So, how can we properly treat this new form of oral disease called peri-implantitis?

## 3 THE ROLE OF THE PERIODONTIST

### 3.1 Diagnosis

As periodontists, we were always called upon to know how to treat our patients in the most appropriate way by recognizing the importance of proper prevention, diagnosis, management and health care of periodontal tissues.

The evaluation and stratification of risk class is to be performed in the same way in patients with implants. Risk factors such as plaque accumulation, smoking, or other systemic health problems such as auto-immune diseases [7, 8] and the use of some drugs which determine gingival hyperplasia are common risk factors for both the periodontal and implant treatment. In addition, eligible patients for implant therapy can greatly benefit from a periodontal treatment that is aimed at supra and subgingival plaque control, especially if this regimen is established prior to implant placement, so as to achieve good levels of oral hygiene that are essentials for a successful outcome of the following implant rehabilitation.

### 3.2 The Role of a Supportive Periodontal Treatment

The approach that includes supportive care is even more important in patients undergoing implant-prosthetic therapy because recent evidence indicates that these patients would mostly benefit from proper management and maintenance of periodontal tissues [8]. At the same time, most patients, generally, have no concept of the importance of a proper management demanded by an implant placement. This class of patients is rarely informed about the real duration of implants and the relative supported prostheses and is sometimes not well or constantly followed and included in the correct maintenance protocols aimed at obtaining adequate plaque control in the long term.

A 30-year follow-up study has shown that systematic maintenance of plaque control can prevent the loss of periodontal and bone tissue in patients that are involved in periodontal supportive therapy, regardless of their age [6].

This study also demonstrated that patients with an average age of 60 to 80 years, included in a constant periodontal supportive protocol (both professional and home maintenance), over 30 years of follow-up, lose an average of 0.7 teeth per subject [6]. These results clearly show that those individuals encouraged to maintain high standards of oral hygiene that have been included in specific supportive care regimens at regular intervals, showed a much lower incidence of both periodontal disease as well as a lower percentage of tooth loss.

It has been widely shown that patients undergoing implant treatment included in maintenance programmes benefit more than those who are not included, or who do not follow consistently this programme [6, 7]. Although peri-implantitis and implant loss are the main eventualities to be taken into account in this type of treatment, there is the possibility that there are also other minor complications such as marginal bone loss and peri-implant mucositis, all pathologies that must be prevented as soon as possible.

Sometimes, the real status of peri-implant disease and the complications of the soft peri-implant tissues may not be well estimated. This can be due to the differences in diagnosis between the different operator during the examination of the peri-implant tissues or due to the lack in the precision of the peri-implant probing or differences in the peri-implant bone loss evaluation at X-ray [7]. Furthermore, the implant treatment prognosis is often reported as the percentage of survival, in which it was up to 95% of reported cases [6, 7]; however, the term “survival” takes into account only the implant permanence and does not adequately describe the total health status of the peri-implant tissues of support. The term “survival” includes not only the state of “peri-implant mucositis”, which is a reversible lesion only circumscribed to soft tissues but also includes the state of “peri-implantitis”, which, instead, is a lesion of bone tissue that, if not properly treated, can lead to the complete loss of the implant [9-11]. These observations can be reflected in the literature by analyzing the wide ranges of values ​​reported for the prevalence of peri-implant mucositis (8% - 46%) and peri-implantitis lesions (up to 25%) [10-13]. An explanation of the diverse and extensive range recorded between the different studies [11, 12] can be explained by variances in the definition and proper diagnosis of these two clinical conditions different from each other but which have similar clinical symptoms.

It is therefore predicted that the frequency of peri-implant lesions and disease will surely increase over the years due to the number of implants used and consequently a major number of studies with more long-term follow-up and the implant survival rate will also change according to the age in which patients undergo these procedures. The same type of surgical procedures should also become even more less invasive. But what does this mean for the increased use of implants in our patients? How then should we define the success of implant therapy?

Without doubt, the development of implantology was a great help and a step forward for prosthetic rehabilitation and allowed oral rehabilitation types never conceived before. The possibility of use of implants provided benefits especially for patients who presented removable prosthetic solutions that gave unsatisfactory aesthetic and functional results in both the medium and long term period. After the advent of implantology, today the main problem could be a possible inappropriate approach and abuse of such a solution, including cases in which a conservative approach could be the best form of therapy.

The main problem is that sometimes implant solutions, which are suggested by clinical and industry experts, are advertised as fast, simple and without risk and as “miraculous” solution for replacing “hopeless” teeth. Until now, no system has proven to be more durable than a natural tooth [14, 15]. Thus, any non-extractive treatment that aims primarily at dental element preservation should be considered before tooth substitution with implant placement [16, 17].

During the last few years new and interesting advances have been developed in periodontal treatment such as regenerative therapy, growth factors and host immuno-modulation treatment [17, 18], all tools useful for tooth maintenance [18-20]. In the rush to adopt implant therapy as the most rapid and effective, these new possibilities of therapeutic approach must not be ignored, because their importance both for periodontology and implantology will definitely increase over the years.

The objective is to select those patients who would benefit from this type of approach compared to everyone else and that a strict stratification of patient risk can be crucial in the decision to approach the patients with implant procedures. Rigorous risk modelling can be useful in such decisions. Different possibilities exist because some patients may be classified to be at such a low risk of tooth loss that annual preventative care for them is useless or at least with a preventable economic cost [20, 21].

It was demonstrated that a prophylaxis specifically directed at primary and tertiary prevention of periodontal and peri-implant disease in the adult together with caries prevention, dental malocclusions and therapies of impacted tooth are one of the most used support services in the world by different national health systems [22-24]. Dental visits have an annual cost of about $ 500 million, which accounts for 76% of the total cost of dental services in the US [25].

The detailed results of the 11^th^ European workshop on Periodontology specific recommended that an appropriate diagnosis alongside assessment of patient-level factors (risk factor and attitudes) should determine the proper selection of the most appropriate type of professional preventive care [26] The new methods of approach in medicine, such as the stratification of the risk assessment or a personalized diagnosis approach in patients who need therapy are aimed at achieving better results for both patients, who only receive appropriate care and diagnostic investigations, as well as for health systems, because care resources are more wisely used.

### 3.3 The Personalized Periodontology

Personalized medicine integrates genomic and clinical information to predict a possible predisposition and especially the progression of the disease [25, 27-29], as well as attempts to provide a better therapy, using specific drugs and clinical treatment for each patient and only for those patients who need a particular type of an early treatment [30-34].

Giannobile and colleagues, in their original work [35], tried to seek evidence on an important concept, and their pioneering effort in this direction should not be abandoned. The concept is that expensive screening or other interventions can be avoided for many patients by “personalized” (or better termed as “stratified”) approaches.

Following these new intriguing therapeutic approaches, periodontology, in a medical space increasingly dominated by a seemingly new “implantology mind” is called upon to clearly define its role within the scope of the dental field. The good news is that this discipline, at present, has become a highly specialized and technological branch, through an approach that is gradually becoming more and more biomolecular and genomic. Today, the periodontist has the correct and sufficient knowledge to decide how to best treat the patient.

## CONCLUSION

Only a balanced and biological approach seems to be the best route for the appropriate maintenance and care of oral health of our patients.
